# Punctuated Shutdown of Atlantic Meridional Overturning Circulation during Greenland Stadial 1

**DOI:** 10.1038/srep25902

**Published:** 2016-05-19

**Authors:** Alan Hogg, John Southon, Chris Turney, Jonathan Palmer, Christopher Bronk Ramsey, Pavla Fenwick, Gretel Boswijk, Michael Friedrich, Gerhard Helle, Konrad Hughen, Richard Jones, Bernd Kromer, Alexandra Noronha, Linda Reynard, Richard Staff, Lukas Wacker

**Affiliations:** 1 Waikato Radiocarbon Laboratory, University of Waikato, Private Bag 3105, Hamilton, New Zealand; 2 Department of Earth System Science, University of California, Irvine, CA 92697-3100, USA; 3 Climate Change Research Centre, School of Biological, Earth and Environmental Sciences, University of New South Wales, Australia; 4 Research Laboratory for Archaeology and the History of Art, University of Oxford, Dyson Perrins Building, South Parks Road, Oxford OX1 3QY, UK; 5 Gondwana Tree-Ring Laboratory, P.O. Box 14, Little River, Canterbury 7546, New Zealand; 6 School of Environment, University of Auckland, New Zealand; 7 Institute of Environmental Physics, University of Heidelberg, INF 229, D-69120 Heidelberg, Germany; 8 Institute of Botany, Hohenheim University, D-70593 Stuttgart, Germany; 9 GFZ German Research Centre for GeoSciences, Dendrochronology Laboratory, Telegrafenberg, 14473 Potsdam, Germany; 10 Marine Chemistry and Geochemistry, Woods Hole Oceanographic Institution, Woods Hole, MA 02543, USA; 11 Department of Geography, Exeter University, Devon, EX4 4RJ, UK; 12 Department of Human Evolutionary Biology, Harvard University, Divinity Avenue, Cambridge, MA 02138 USA; 13 Laboratory of Ion Beam Physics, HPK, H29, Otto-Stern-Weg 5, CH-8093 Zürich, Switzerland

## Abstract

The Greenland Stadial 1 (GS-1; ~12.9 to 11.65 kyr cal BP) was a period of North Atlantic cooling, thought to have been initiated by North America fresh water runoff that caused a sustained reduction of North Atlantic Meridional Overturning Circulation (AMOC), resulting in an antiphase temperature response between the hemispheres (the ‘bipolar seesaw’). Here we exploit sub-fossil New Zealand kauri trees to report the first securely dated, decadally-resolved atmospheric radiocarbon (^14^C) record spanning GS-1. By precisely aligning Southern and Northern Hemisphere tree-ring ^14^C records with marine ^14^C sequences we document two relatively short periods of AMOC collapse during the stadial, at ~12,920-12,640 cal BP and 12,050-11,900 cal BP. In addition, our data show that the interhemispheric atmospheric ^14^C offset was close to zero prior to GS-1, before reaching ‘near-modern’ values at ~12,660 cal BP, consistent with synchronous recovery of overturning in both hemispheres and increased Southern Ocean ventilation. Hence, sustained North Atlantic cooling across GS-1 was not driven by a prolonged AMOC reduction but probably due to an equatorward migration of the Polar Front, reducing the advection of southwesterly air masses to high latitudes. Our findings suggest opposing hemispheric temperature trends were driven by atmospheric teleconnections, rather than AMOC changes.

Changes in the strength of the AMOC, as defined by the northward transport of surface warm water masses to sites of deep and intermediate water formation, are widely considered to be the major driver of surface temperature trends in the North Atlantic during the termination of the last glacial period (the Lateglacial; 15–11.5 kyr cal BP)^1^,^2^,^3^. Importantly, within the termination, a sustained period of cooling described as GS-1 is recorded in the Greenland ice cores, and thought to be initiated by freshwater runoff from North America and/or Fennoscandinavia^4^,^5^,^6^ (Fig. 1). Associated with GS-1 is a broadly synchronous change in Europe described as the Younger Dryas (YD) stadial, the pollen assemblage zone during which there was a return to near-glacial conditions and coincident with an abrupt change in radiocarbon ages (~12.70 to 11.65 kyr cal BP)^7^,^8^,^9^,^10^,^11^. Contrasting temperature changes inferred from the mid to high latitudes of the Southern Hemisphere (SH)^12^,^13^ have been used as evidence in support of an ocean ‘bipolar seesaw’ for the global redistribution of heat^1^,^14^. Because of relatively large chronological uncertainties of key Antarctic sequences^12^, however, significant debate remains around the timing and origin of North Atlantic cooling during the GS-1^4^,^8^ and the preceding Antarctic Cold Reversal (ACR) in the SH^12^,^15^,^16^, raising the possibility that other mechanisms (e.g. sea ice expansion, atmospheric circulation change)^8^,^17^,^18^ and/or regions (e.g. Southern Ocean or tropics)^19^,^20^ may have played a major role.

Annually-resolved tree-ring sequences provide a means of testing hypotheses of synchronous change through the development of a securely-dated record of atmospheric ^14^C. Unfortunately, a continuous atmospheric record though GS-1 has proved elusive. Work on the European Lateglacial Interstadial (i.e. Bølling-Allerød) and YD pines has generated a series of floating Northern Hemisphere (NH) ^14^C measurements^21^,^22^,^23^,^24^ linked to the Holocene using floating sections of SH Tasmanian huon pine spanning ~620 years of the GS-1 chronozone^22^. The inability to cross-date the huon tree rings and the recent identification of an incorrectly placed 200-year late YD European larch chronology^25^, raises significant doubts over the international calibration curve (IntCal13)^23^ across this period and by implication previous efforts to precisely date abrupt and extreme change across the Lateglacial^4^,^26^,^27^.

Here we exploit sub-fossil kauri trees (*Agathis australis*) recovered from bogs in northern New Zealand^28^ to resolve this impasse. We present a new decadally-resolved record of atmospheric ^14^C from a cohort of 40 sub-fossil kauri logs that have recently been discovered at a farm near Towai (35°30′S, 174°10′E) in Northland, producing a floating 1451-yr tree-ring chronology, supplemented by a single 533-yr tree ring record (FIN11) from Finlayson Farm, near Kai Iwi Lakes, Northland (35°50′S, 173°39′E) which together span the full GS-1 chronozone. Radiocarbon dating by 5 laboratories (Waikato, Irvine, Oxford, Zurich and Heidelberg) on cellulose extracted from wood samples from the Towai chronology and FIN11 has produced 1022 measurements (Supplementary Tables 1 and 2), providing the most comprehensive atmospheric record of ^14^C for the GS-1 thus far (see Methods and Extended Data).

We firstly anchored the floating Towai and FIN11 SH data sets to known-calendar age NH sequences, by comparing the kauri decadal ^14^C measurements with IntCal13, but ensuring that only the robust tree-ring Preboreal Pine Chronology (PPC)^24^ part of IntCal13 was used for the curve matching (see Methods). The combined SH series spans the period 13,134 to 11,366 cal BP with a pronounced rise in Δ^14^C beginning at ~12,800 cal BP, followed by three peaks and a subsequent decline through the GS-1 chronozone into the Holocene (Fig. 2E). The triplet of peaks has been identified previously^22^ but was assigned an incorrect calendar age.

Importantly, the new kauri series, in addition to having a ~480-yr overlap with the PPC, spans or overlaps with two separate, floating, dendrochronologically secure ^14^C-dated NH tree ring series (the 399-yr YD_B pine series^22^,^24^ and the 1606-yr Central European Lateglacial Master (CELM) chronology pine series^21^,^29^), providing the opportunity to develop a continuous, securely-dated record of atmospheric ^14^C through GS-1. The YD_B ^14^C series maps onto the ~10,400 ^14^C BP plateau as recognised in the kauri (Figs 1B and 2D) and indicates this period of near-constant radiocarbon age in the YD as reported in IntCal13^23^ is ~50 cal years too short (Extended Data Fig. 3). Our positioning of the CELM and YD_B data sets is consistent with other terrestrial NH ^14^C data, including the U-Th series dated Hulu Cave H82 speleothem^30^ (Fig. 1B, Extended Data Figure 4), giving confidence in our precise calendar placement using the kauri and providing the first continuous atmospheric record extending back to 14,174 ± 3 cal BP.

To put the atmospheric radiocarbon variations in the context of climate and ocean changes in the northern Atlantic, we compared kauri tree-ring atmospheric ^14^C with surface marine ^14^C obtained from the tropical annually-laminated Cariaco Basin (10°43′N, 65°10′W) which is securely dated via ^14^C comparisons with early Holocene NH tree ring series^31^. Before ~12,920 and after ~12,640 cal BP we observe coherence between the ^14^C records of Cariaco and the kauri (Figs 2 and 3) including matching individual peaks in radiocarbon age (e.g. 12,500 cal BP) indicating that the varved marine sequence is robust. However, we observe a dramatic collapse in the ~420 ^14^C yr marine reservoir age (causing a > 2 sigma divergence between reservoir corrected Cariaco and atmospheric ages) commencing at ~12,920 cal BP (Figs 1C and 3). Reservoir-corrected Cariaco Basin and Atlantic coral data do not re-converge with the kauri ^14^C until ~12,640 cal BP (Figs 1C and 3). In marked contrast, reservoir-corrected Pacific Ocean coral ages map onto the atmospheric kauri record^23^. Previous workers have suggested that a dramatic collapse/reduction in AMOC as a result of freshwater hosing in the North Atlantic during the onset of GS-1 and Heinrich Stadial 1 (HS1)^2^,^4^ led to increased stratification and enhanced air-sea mixing causing younger surface waters in the subtropical North Atlantic^30^. We therefore interpret divergence between the reservoir-corrected Cariaco and Towai records as a proxy for reduced AMOC.

Cariaco also provides a sensitive measure of latitudinal changes in the trade winds associated with the Intertropical Convergence Zone (ITCZ); where an ITCZ migration to the south enhances upwelling and therefore productivity along the coast resulting in lighter-coloured biogenic laminations^31^. A sustained shift to lighter greyscale values accompanies the drop in reservoir ages ~12,920 cal BP, but importantly, with the convergence of marine-atmosphere ^14^C at ~12,640 cal BP (implying renewed AMOC strength), the ITCZ remains to the south (Fig. 3).

The new atmospheric radiocarbon calibration curve provides a securely anchored timescale for determining the timing and sequence of events through GS-1. Our results agree with ^231^Pa/^230^Th and ^143^Nd/^144^Nd studies in marine cores^2^,^32^ that describe a brief sharp decline in AMOC immediately followed by a gradual re-acceleration in the GS-1, but provide a considerably better dated and more highly-resolved record that is not subject to bioturbational smoothing. The record of atmospheric ^14^C concentration presented here suggests a decline in the AMOC for ~290 years, beginning at ~12,920 cal BP, and an additional though smaller ~150 year divergence of the marine (corrected) and atmospheric ^14^C ages between 12,050 and 11,900 cal BP (Fig. 2E). The timing of the initial slowdown in the AMOC is synchronous within chronological uncertainty with the onset of Greenland cooling following GI-1a at 12,875 ± 59 cal BP^19^,^27^, weakening of the Asian Summer Monsoon recorded in U/Th dated Hulu Cave speleothem H82 at 12,950 ± 50 cal BP^30^ and the termination of the Allerød interstadial in the varved Meerfelder Maar sequence at 12,890 ± 31 cal BP (aligned with the Towai record by determining a ^14^C wiggle match age for the Laacher See Tephra of 12,893 ± 3 cal BP-see Methods)^8^,^33^. Furthermore, the resumption of the AMOC after ~12,640 cal BP as identified by the atmospheric-marine ^14^C comparison is synchronous with maximum cooling in Greenland at 12,662 ± 74 cal BP^19^,^27^ and increased aridification and cooling plus major vegetation changes marking the onset of the European YD as recorded in the varved Meerfelder Maar sediment record at 12,720 ± 40 cal BP^8^. In contrast to recent modelling work arguing for sustained freshwater hosing^3^,^34^, the onsets of our two inferred reductions in AMOC are coincident, albeit within significant uncertainties, with two ^14^C-dated peaks of freshwater flux into the North Atlantic via the St Lawrence River^4^ recalibrated here at 13.1 ± 0.1 kyr cal BP and 12.1 ± 0.2 kyr cal BP (Fig. 2). Hence, while our results support freshwater input as the primary cause of the slowdown of AMOC, it does not appear to have driven cooling across the full GS-1 as recorded in Greenland^32^,^35^.

A major advantage of a Southern Hemisphere record of atmospheric ^14^C is that it can inform on past sources of CO_2_ (ref. 36), helping to resolve the timing of events in both hemispheres. Crucially, deep water formation in the North Atlantic isolates surface water, most of which upwells in the Southern Ocean, induced by the strong, persistent westerly winds in the SH^37^. Rapid overturning precludes full re-equilibration with atmospheric CO_2_, and renewed isolation within the deep Pacific provides sufficient time for radioactive decay to significantly reduce ^14^C activity of the abyssal waters, resulting in Southern Ocean upwelling (and outgassing) of old CO_2_ (refs 36,38), depleting atmospheric ^14^C levels and causing ‘modern’ SH samples to be ~35 ^14^C yrs older than their NH counterparts^38^.

Thus, comparison between the SH kauri and NH pine datasets provides an opportunity to investigate the atmospheric interhemispheric ^14^C gradient (IHG) as a measure of Southern Ocean ventilation during the GS-1 chronozone^38^. We observe a remarkable near-zero gradient between the hemispheres during the Lateglacial Interstadial (Fig. 1E). Regime shift analysis of the IHG dataset using 90% confidence^39^ suggests that at ~12,660 cal BP, the SH offset assumed near-modern values (see Methods). Determining the magnitude of the ^14^C gradient precisely requires coupled analyses of contemporaneous NH and SH sample pairs within the same laboratory, but based on two lines of evidence we consider it unlikely that the low ^14^C gradient for periods older than ~12,660 cal BP is a result of inter-laboratory differences (see Methods).

Regardless of the exact magnitude of the ^14^C offset, the observed shift to older SH ages is consistent (and synchronous within the uncertainty in the WAIS Divide chronology across this period^12^–see Fig. 1G) with increased ventilation of the Southern Ocean following the termination of the ACR, associated with reinvigoration of Antarctic Bottom Water formation, sea ice retreat and reduced Antarctic ice melt^15^,^40^,^41^,^42^. It is important to note that Southern Ocean ventilation appears to have recommenced almost simultaneously with AMOC recovery in the North Atlantic and was sustained through the GS-1 and into the Holocene (Fig. 1E). Recent work has postulated a reorganisation of atmospheric circulation during maximum North Atlantic cooling, leading to the strongest expression in European sequences defining the YD^8^. Given the synchronous nature of the southernmost extent of the ITCZ^31^ (Fig. 1D) and SH westerlies^42^,^43^ at ~12,660 cal BP, our results suggest the parallel increases in Southern Ocean ventilation and Antarctic warming^12^,^13^ may have been driven by atmospheric forcing, rather than an ocean bipolar seesaw^1^.

The mechanisms responsible for cooling across Europe and Greenland while the AMOC intensity increased during the GS-1^19^,^26^ remain unclear, but one possibility is that the first freshwater event at the onset of this event led to a southerly migration of the Polar Front in the North Atlantic^5^,^8^,^44^ and that this equatorward shift was maintained after the AMOC was reinvigorated. Sustained frigid conditions post 12,640 cal BP and a postulated reorganisation of atmospheric circulation^8^,^45^ are coincident with the triplet of peaks identified in both tree ring Δ^14^C and ^10^Be in Greenland ice^46^,^47^,^48^ (Fig. 2) suggesting periods of reduced solar activity may have played a similar role to those that occurred during the Little Ice Age (LIA; CE 1580–1880)^49^,^50^,^51^. The more extensive sea ice cover in the North Atlantic from 12,900 cal BP onwards, implied by the Greenland record^19^,^52^, may have resulted in increased sensitivity to LIA-type centennial solar minima through this period. Modelling studies have shown that reduced solar UV fluxes can result in more negative North Atlantic Oscillation-like conditions^53^, with a weakening of the Icelandic Low and hence a reduction in southwesterly airflow over Europe, driving regional cooling. If similar atmospheric dynamics were present and climate sensitivity was enhanced in a period where the AMOC was reduced or absent and sea ice extended to lower latitudes, the resultant cooling south of the ice front may have been sufficient to restart convection, but in a southward-shifted “glacial-cold” mode centered on the northeast Atlantic rather than the Nordic seas^44^. Thus, even if northward heat transfer associated with the reinvigorated AMOC reached near-Holocene levels^32^, the polar front was effectively pinned in a southerly position that left much of Europe out in the cold. Regardless of the precise mechanism^3^, the identified convergence in atmosphere-marine ^14^C and the establishment of a ‘modern’ IHG between 12,660 and 12,640 cal BP strongly argues against changes in AMOC as the sole cause of GS-1 cooling in the NH. Our results support a growing body of evidence that global scale changes during the GS-1 chronozone^3^,^16^ were primarily driven by interhemispheric atmospheric teleconnections.

## Methods

### Dendrochronology

A master tree-ring chronology was compiled from a cohort of 40 Towai sub-fossil kauri logs^25^ (Extended Data Fig. 1). The Towai sub-fossil kauri floating tree-ring chronology was compiled from 91 radial strips and is well replicated and securely cross-dated with an average cross-correlation coefficient between all series of 0.71. To compensate for the inadequate sample depth of the youngest 164 rings^25^, and to investigate the possibility of a significant shift in the radiocarbon interhemispheric gradient (IHG) at the end of the Younger Dryas stadial, we obtained the kauri log FIN11 from Finlayson’s Farm at Kai Iwi Lakes, near Dargaville (Extended Data Fig. 1), which overlaps with the Towai chronology by ~185 years and extends the kauri measurements a further ~290 years into the Early Holocene. The tree FIN11 has two measured radii and 533 rings. Although the exact number of years represented cannot be precisely known because it is only a single tree, the average number of missing rings for New Zealand kauri is very low (<1%) and false rings are rare^28^,^54^.

### Wood pretreatment and ^14^C measurement

Radiometric liquid scintillation (LS) spectrometry and gas proportional counting and accelerator mass spectrometry (AMS) radiocarbon dating have been undertaken on cellulose extracted from decadal wood samples from both the Towai chronology and FIN11. Detailed wood pretreatment procedures and ^14^C analytic methods for the 3 principal participating labs (Waikato University-Wk, University of California at Irvine-UCI, Oxford University–OxA) are given elsewhere^25^. The number and method of analyses varied between labs because two labs (ETH Zurich-ETHZ, and University of Heidelberg-HD) dated only a few consecutive decadal samples as part of an inter-laboratory comparison^25^ or the amount of wood available was only sufficient for AMS dating. Contributions from the 5 labs are as follows:

(a) Towai chronology (145 decadal samples; 778 analyses). UCI AMS; holo-cellulose; 144 decades; Wk LS spectrometry; α-cellulose; 117 decades; Wk AMS; α-cellulose; 4 decades; OxA AMS; α-cellulose; 117 decades; ETHZ AMS; α-cellulose; 12 decades; HD gas proportional counting; holo-cellulose; 10 decades.

(b) FIN11 (48 decadal samples; 244 analyses). UCI AMS; holo-cellulose; 48 decades; Wk AMS; α-cellulose; 45 decades.

### Statistical analysis of ^14^C data and computation of decadal means

High precision and accuracy was accomplished through high replication of decadal samples using different dating approaches and numerous participating laboratories. Any result for which the ^14^C age plus or minus 2.576 times the 1σ error did not enclose the median value was rejected as an outlier. This 2.576 sigma corresponds to the range within which 99% of samples should lie; as a rejection algorithm this should reject ~1/100 values. Error weighted mean values (X_mean_) and associated errors (E_stat_) were calculated for the accepted results for each decade. To calculate a standard error that takes into account the dispersion of the individual results, we calculated E_std_ with the final error (E_final_) the larger of E_stat_ and E_std_^38^. Decadal error weighted mean ages and errors, the number of analyses accepted and rejected per decade, and χ2 agreement values are given in Supplementary Data Table 1 (Towai chronology) and Table 2 (FIN11). From a total of 1035 results, 11 were rejected from the Towai chronology and 2 from the tree FIN11. The χ2 agreement indices indicate a high level of reproducibility within each decade for both data sets and this is augmented by the use of the conservative E_final_ standard error term, which reflects internal variability. The final data sets presented show that the 3 main participating laboratories (UCI, Wk and OxA) are highly consistent with very low inter-laboratory offsets (Wk-UCI = 0.2 ± 2.6 yr; Wk-OxA = −3.8 ± 4.8 yr; and UCI-OxA = −3.4 ± 4.1 yr), justifying the approach taken here.

### Assignment of calendar ages by ^14^C Bayesian curve matching

We anchored the two floating SH kauri data sets Towai and FIN11 by comparing kauri decadal ^14^C measurements to IntCal13^23^, but ensuring that only the robust tree-ring Preboreal Pine Chronology (PPC) part of IntCal13 was used for the curve matching (see ‘Calendar placement of Towai and FIN11 data sets’ section below). We then anchored the floating Late Glacial NH tree-ring series YD_B and CELM (see details below) by matching published NH ^14^C data^21^,^22^,^24^,^29^ with the secured Towai and FIN11 ^14^C data sets. We utilised the D_Sequence function of OxCal 4.2 and applied the OxCal Reservoir Offset (‘Delta_R’) function^55^ with a uniform prior to account for the ^14^C Interhemispheric Gradient (IHG). We also employed outlier analysis, using Outlier_Model ("SSimple",N(0,2),0,"s") and with a prior outlier probability of 5% applied to each ^14^C measurement (‘{Outlier, 0.05}’) to identify and down-weight statistical outliers^56^. The matching of the data sets occurred in 2 stages. The first step utilised a wide uniform prior for the IHG of −120 to + 120 yrs (*i.e*., Delta_R = (U(−120,120)), to identify the most probable calendar positioning of the time series. A wide prior was chosen for this initial screening to accommodate possible extreme changes in the IHG that may have resulted from postulated ocean circulation changes. The second step used the same wide uniform Delta_R prior but was restricted to the most probable calendar positioning as indicated in step 1 and provided a more precise calendar age range. The OxCal agreement index A_comb_ together with χ^2^ agreement data indicates the quality of fit between the various data sets. For acceptable agreement in the context of curve matching, A_comb_ should be significantly higher than A_n_ (*i.e*. 1/**√**2n), where n equals the number of observations in the floating data set.

### Calendar placement of Towai and FIN11 data sets

The extended ‘absolute’ NH tree-ring data set incorporated into IntCal13 although reported as extending to 12,594 yr cal BP^22^ has large uncertainties and low sample density around ~11,900 cal BP (~10,260 ^14^C yr BP) as a result of the removal of the Ollon (VOD) 505 dataset^25^. Matching a floating Lateglacial series against IntCal13 must therefore use ^14^C data points younger than ~10,260 ^14^C yr BP. The Towai chronology contains 18 appropriate decadal data points and these have been matched to IntCal13 using a uniform reservoir offset function prior of U(−120,120))–Extended Data Fig. 2. There is high agreement for both individual analyses and the model as a whole (model agreement index A_comb_ = 262.3%) with the youngest decade having a mean calendar age of 11,694 ± 7 cal BP. Using this fit, the Towai sequence decadal mid-points lie between 11,694 and 13,134 cal BP. The Finlayson Farm tree FIN11 was analysed to provide confidence that there were few missing Towai rings and to provide a more robust lock with the Late Glacial/Early Holocene dendro-dated wood series forming IntCal13. All 48 FIN11 decades were matched against IntCal13 using the same Delta_R uniform offset prior of −120 to 120 yr. The model as a whole shows very good agreement (A_comb_ = 298.7%) with the youngest FIN11 decade having a mean calendar age of 11,366 ± 3 cal BP. The FIN11 decadal mid-points therefore range from 11,366–11,869 cal BP (Extended Data Fig. 2). FIN11 data agree well with the Towai ^14^C series in the region of overlap (A_comb_ = 91.9%; χ2-test: *T* = 16.3 (5% 27.6)). The two matches as outlined above have produced decadal mid-point ranges of 11,694–13,134 cal BP (Towai) with a 1σ error of ± 7 yr and 11,366–11,869 cal BP (FIN11). The overlap with IntCal13 is ~480 cal yrs. We have not assigned an error to the FIN11 calendar age range because of the possibility of some missing rings, though the number of these is probably very small (see Dendrochronology). However, the FIN11 ^14^C time series does confirm that there is no measurable change in the IHG at the beginning of the Holocene. It should be noted that the primary purpose of the FIN11 ^14^C series was to provide added confidence in the placement of the Towai dataset to IntCal13; the younger FIN11 data were not used in the alignment of the floating NH datasets, YD_B and CELM.

### Refinement of the Northern Hemisphere Late Glacial tree-ring series

The Lateglacial and early Holocene radiocarbon record, as reported in IntCal13, has been compiled through mostly decadal ^14^C dating of tree-rings from three key chronologies: the ‘absolute’ tree-ring chronology starting at 12,410 cal BP, with the oldest section represented by the Preboreal pine chronology (PPC) dendro-linked to the Hohenheim Holocene oak chronology^24^; extension of the absolute tree-ring chronology by 184 yr to 12,594 cal BP utilising Swiss pines in a chronology called YD_B, dendro-matched to the earliest part of the PPC^22^; and the floating 1606-yr Central European Lateglacial Master Chronology–CELM^29^ extending the YD_B chronology to ~14,000 cal BP^21^,^29^. Importantly, the new kauri series, in addition to having a ~480-year overlap with the PPC, spans or overlaps with both the YD_B pine series, spanning 399 years^22^ and the 1606-yr Central European Lateglacial Master (CELM) chronology pine series^21^,^29^. The 1451-year long Towai kauri sequence provides sufficient detail in atmospheric radiocarbon to precisely place these NH pine ^14^C sequences against calendar time.

Comparison with YD_B (the dendrochronologically secure, Swiss, 7-tree, 399-yr long, tree-ring series including Gänziloh trees G22, G27, G34 and G102, Birmensdorf B200 and the two Zurich trees KW30 and 31–refs 22,29) provides two possible placements for the NH sequence (Extended Data Figure 5), with the more probable solution generating an agreement index of 203% and the youngest part of the series dated to 12292 ± 5 cal BP; the other possible solution we discount here as it has a relatively low agreement index (63%) and data points that are misaligned with the oldest PPC measurements. The CELM ^14^C data set was compiled from German and Swiss chronologies^21^,^29^ which provide 232 ^14^C dates, covering the radiocarbon age interval of 10,612–12,357 ^14^C yr BP, contained within IntCal13 (ref. 23). It should be noted that the youngest sample from the CELM chronology in the IntCal13 data set, is from tree Gänziloh 3 (G3: HD-22487). This sample is 11.5 cal yr younger than the youngest sample (Gaen5: HD-22482) from the 2004 LGP chronology^21^. The youngest 72 CELM ^14^C measurements (from the IntCal13 database (http://intcal.qub.ac.uk/intcal13/) overlap with the Towai data set and were compared with it using a uniform Delta_R prior of -120 to + 120 yr. Despite the wide uniform prior, the CELM has only one possible solution against the Towai kauri ^14^C record as a result of the rapid decline in atmospheric radiocarbon ages beginning ~12,750 cal BP (Extended Data Figure 6), producing an agreement index of 480% and placing the youngest age in the sequence (G3: HD-22487) at 12,606 ± 3 cal BP, with a mean IHG of 3.1 ± 3.6 yr. This positioning places the youngest of the LGP^21^ samples (Gaen5, HD-22482) at 12,618 ± 3 cal BP. This calendar age is statistically indistinguishable from the IntCal13 placement and agrees with some studies^22^,^57^ but disagrees with others^58^. The region of overlap between the CELM and Towai data sets agree reasonably well, with the overlap between the data sets shown in Extended Data Figure 6.

We observe that IntCal13 for the YD interval contains two principal errors. Firstly, the curve is too young for the interval 11,860 to 12,150 cal BP, which previously contained the Ollon505 series (Extended Data Fig. 3) and for which no replacement NH data are yet available. Secondly, the ~10,400 ^14^C yr plateau is ~50 cal yrs too short (Extended Data Figure 5).

### Calendar age assignment for Cariaco Basin radiocarbon dates

Both the Cariaco and kauri ^14^C series are anchored in calendar time by matching against essentially the same NH tree ring data set. Cariaco varved data achieve a very secure lock with an overlap with IntCal data of ~1900 yr. The matching details are given elsewhere^31^. We used OxCal to check the match of the Cariaco data against the secure part of IntCal13 and obtained a youngest Cariaco calendar age of 10,493 ± 7 cal BP, which is statistically indistinguishable from the published age of 10,503 ± 16 cal BP^31^. We have therefore used the published^31^ calendar age scale in this paper.

### Comparisons with the Meerfelder Maar (MFM) sequence

To synchronise the Towai and MFM timescales, we obtained an accurate and precise calendar age for the Laacher See Tephra (LST), one of the tephras used to anchor the floating MFM varve chronology^8^. The LST occurs 30 varve years before (i.e. 30 years older than) the onset of the decline in biomarker δD values at MFM^8^. We matched a series of ^14^C dates of known calendar spacing obtained for wood (tree Kruft9)^59^ buried by the LST^8^ against the Towai data set using a uniform prior of −120 to + 120 yrs. We obtained a calendar age for the LST of 12,893 ± 3 cal BP, which is statistically indistinguishable from with the MFM-derived age of 12,880 ± 40 yrs^8^. This synchronisation of the two timescales provides additional confidence when comparing the palaeoclimatic proxies from the two data sets.

### Magnitude of the ^14^C Interhemispheric Gradient during the Lateglacial

**Figure 1:**
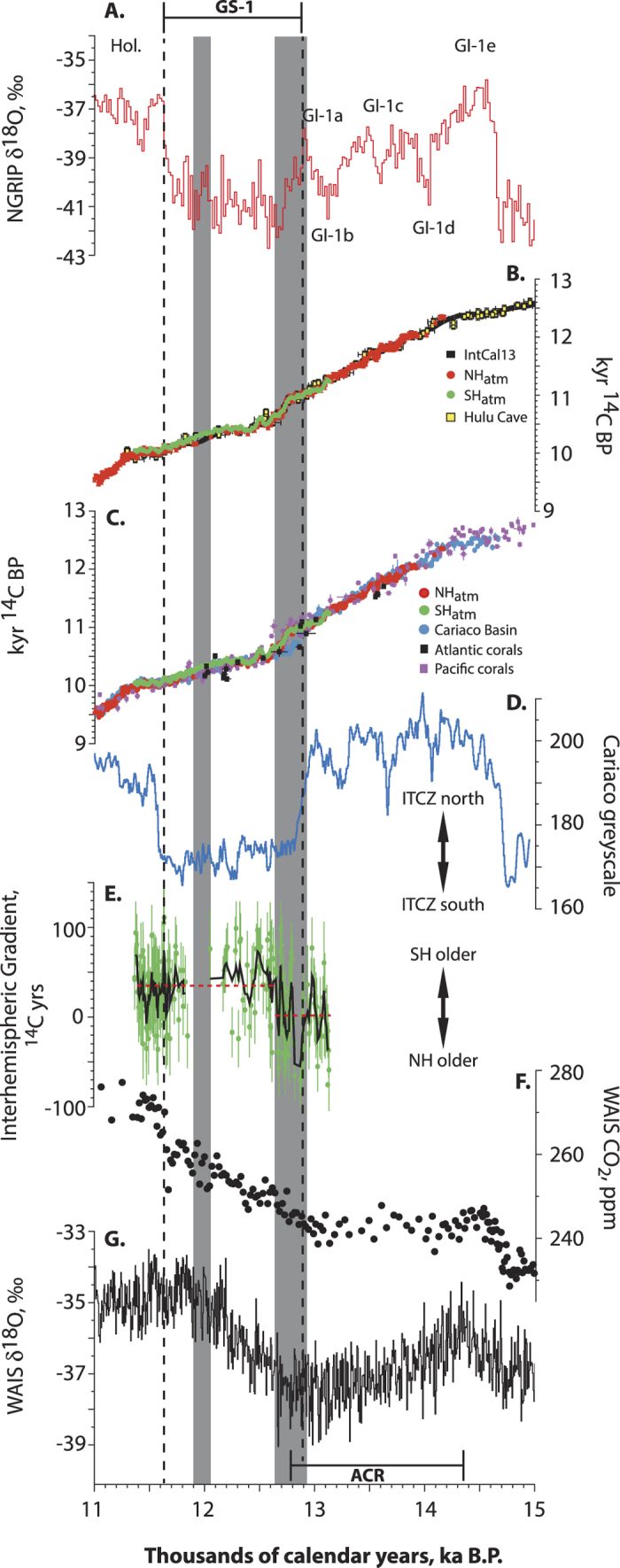
Comparison between Greenland δ^18^O (**A**)^19^, atmospheric radiocarbon datasets^21^,^22^,^24^,^29^,^30^ (**B**), ocean radiocarbon datasets (marine reservoir corrected) expressed as age^23^,^31^ (**C**), Cariaco Basin greyscale^31^ (**D**), the Interhemispheric Gradient with 5-point running mean (solid black line) and mean values during the two identified regimes (dashed red lines) (**E**), atmospheric CO_2_ concentration (**F**) and δ^18^O (**G**), West Antarctic Ice Sheet (WAIS) Divide^12^. Greenland interstadial and stadial events (GI and GS respectively) are shown on **A**^19^. Dark grey columns denote significant slowdown/shutdown of AMOC within GS-1 (divergence between atmospheric and Atlantic marine ^14^C ages >2 sigma); the dashed lines define the GS-1 chronozone^19^. Note, the uncertainty in the WAIS Divide chronology during the termination of the Antarctic Cold Reversal (ACR) around 12,800 cal BP is ± 240 years^12^. All error bars denote 1σ. The gap in the IHG record (**E**) is due to an absence of NH data.

The radiocarbon IHG is the difference in ^14^C age between the two hemispheres and is primarily caused by the larger expanse of SH oceans and the presence of ‘old’ upwelled deep water plus higher wind speeds causing more ^14^C-depleted CO_2_ to enter the atmosphere via air-sea gas exchange^38^. Although the spacing between the NH and SH curves (the IHG) shows variability over the past 2kyr^38^,^60^, the actual calendar positioning of the peaks and troughs is identical. This is an important consideration when matching floating data sets, which may be derived from time periods that experienced different IHG levels. We calculated individual IHG values (SH minus NH) for each PPC, YD_B, and CELM tree-series ^14^C data point. We first constructed a Towai/FIN11 curve with annual resolution, generated by linear interpolation of the Towai/FIN11 decadal values. Having determined a calendar age and associated ^14^C date for each NH data point, we were then able to identify the equivalent SH ^14^C age, with the IHG calculated by subtracting the NH ^14^C age from its SH equivalent. There is a clearly defined decrease in the gradient coinciding with the progression from the PPC and YD_B dates to the CELM dates (SH minus NH, 49 to ~3 yrs), with the change occurring at ~12,660 cal BP (Fig. 1E). Regime shift analysis was undertaken using a sequential algorithm method described and available at http://www.beringclimate.noaa.gov/regimes/39. The PPC and YD_B data sets have a weighted mean IHG value of 38.3 ± 6.0 yr, which is statistically indistinguishable from 2^nd^ Millennium AD values (41.3 ± 1.9 yr) calculated directly from measured SH and NH contemporaneous sample pairs. The IHG for the time interval ~12,600–13,100 yr cal BP is clearly anomalous compared with typical values for younger time periods, including the Holocene. As the IHG values calculated for this study have been derived from ^14^C measurements made by different laboratories (Wk/UCI/OxA for the SH and HD for NH measurements), we have considered the possibility that the apparent reduced IHG levels for periods older than ~12,660 cal BP are the result of inter-laboratory differences. However, we believe this is unlikely, because in two separate inter-laboratory comparisons^25^ HD data were on average 15–20 yrs younger than Wk/UCI/OxA measurements: hence the calculated IHG values presented in this study may be conservative, and the pre-12,660 cal BP values could have been even lower than reported here. Furthermore, a comparison of HD lab numbers for YD_B (normal IHG) and CELM (reduced IHG) shows the majority of these samples were analysed in the HD lab about the same time, strengthening the argument that the reduction in the IHG for the CELM samples is not due to varying HD lab biases.

## Additional Information

**How to cite this article**: Hogg, A. *et al.* Punctuated Shutdown of Atlantic Meridional Overturning Circulation during Greenland Stadial 1. *Sci. Rep.* **6**, 25902; doi: 10.1038/srep25902 (2016).

## Supplementary Material

Supplementary Information

## Figures and Tables

**Figure 2:**
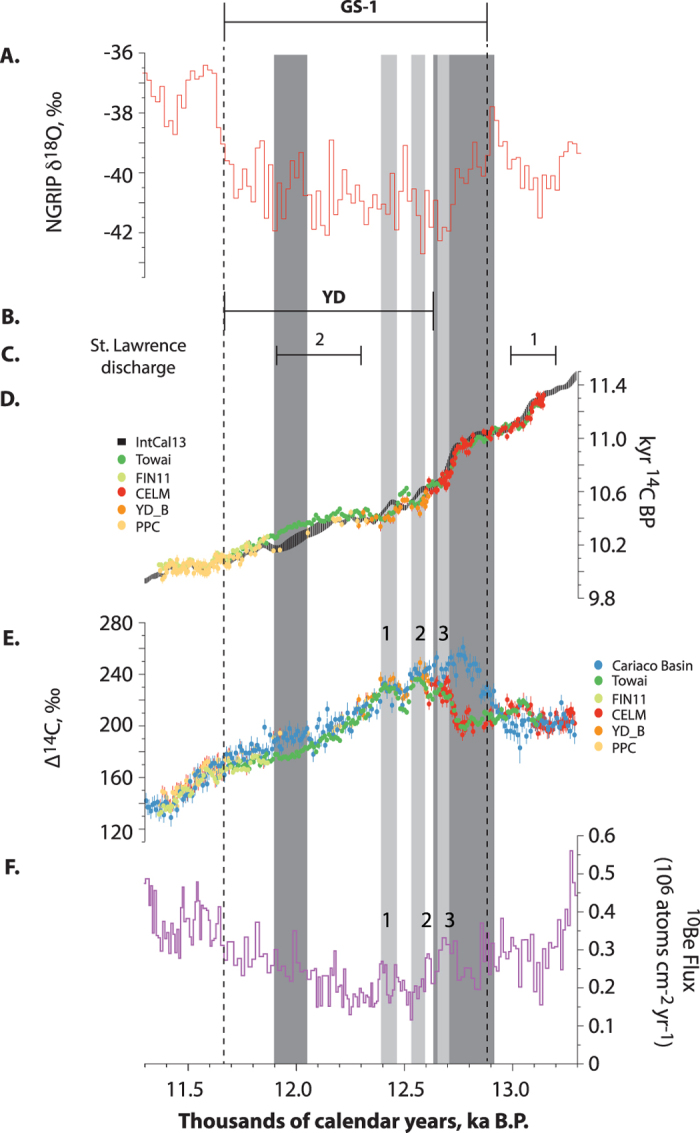
Comparison between Greenland climate and key ^14^C and ^10^Be datasets across GS-1. Greenland δ^18^O (**A**)^19^ with the age range for YD as reported from western Europe^8^ (**B**), recalibrated ages for freshwater fluxes from the St Lawrence River^4^ (**C**), atmospheric radiocarbon datasets^21^,^22^,^24^,^29^ (**D**), Cariaco radiocarbon datasets (marine reservoir corrected) expressed as Δ^14^C (ref. 31) (**E**), and ^10^Be flux in the Greenland ice core^46^ (**F**). Dark grey columns denote significant slowdown/shutdown of AMOC within GS-1 (divergence between atmospheric and Atlantic marine ^14^C ages >2 sigma); light grey columns identify peaks in Δ^14^C with possible peaks (numbered) in ice core ^10^Be (ref. 46). All error bars denote 1σ.

**Figure 3:**
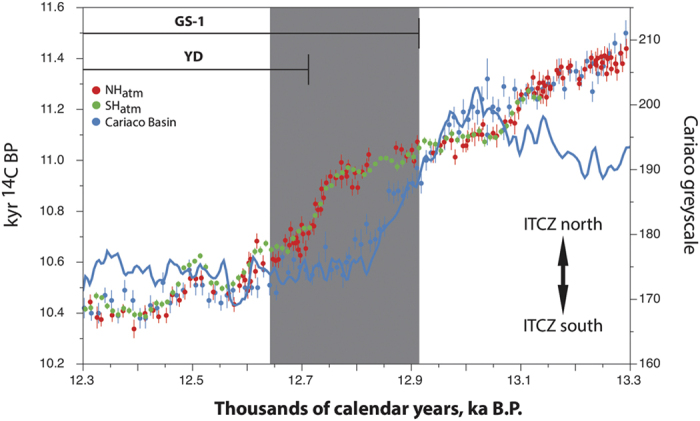
Comparison between atmospheric (including New Zealand kauri) radiocarbon datasets^21^,^22^,^24^,^29^, Cariaco Basin radiocarbon (marine reservoir corrected) and greyscale^31^ datasets. Dark grey column denotes significant slowdown/shutdown of AMOC within GS-1 (divergence between atmospheric and Atlantic marine ^14^C ages >2 sigma). The onset of the YD is as defined in Meerfelder Maar^8^. Latitudinal migration of the Intertropical Convergence Zone (ITCZ) over the tropical Atlantic as inferred by the Cariaco Basin greyscale is also shown.
