# Supplementary material for: Punctuated Shutdown of Atlantic Meridional Overturning Circulation during Greenland Stadial 1
Source: Sci Rep. 2016 May 19;6:25902. doi: 10.1038/srep25902 (PMC4872135; doi:10.1038/srep25902)
Supplement: Supplementary Information [file srep25902-s1.doc]

Extended Data

**Punctuated Shutdown of Atlantic Meridional Overturning Circulation during Greenland Stadial 1**

Alan Hogg*, John Southon, Chris Turney*, Jonathan Palmer, Christopher Bronk Ramsey, Pavla Fenwick, Gretel Boswijk, Michael Friedrich, Gerhard Helle, Konrad Hughen, Richard Jones, Bernd Kromer, Alexandra Noronha, Linda Reynard, Richard Staff, Lukas Wacker.

*To whom correspondence should be addressed. E-mails: [alan.hogg@waikato.ac.nz](mailto:alan.hogg@waikato.ac.nz) and c.turney@unsw.edu.au

**
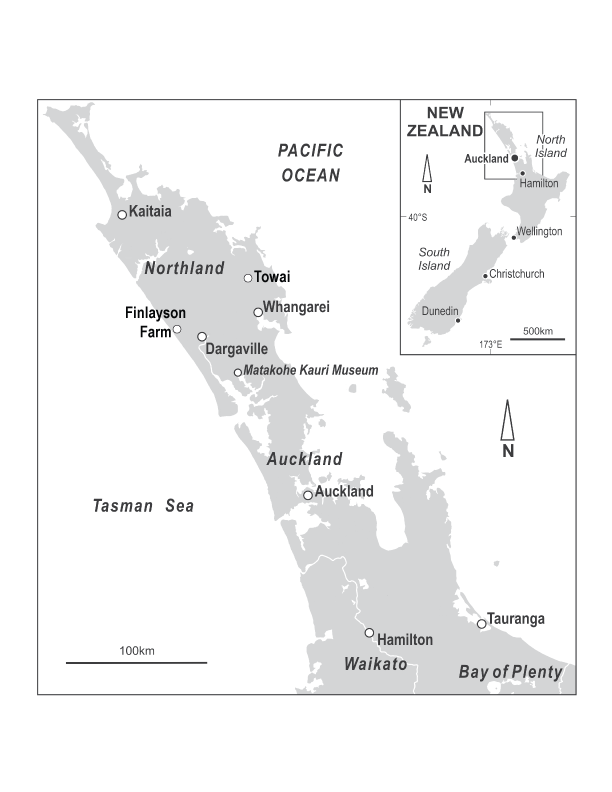
**

**Extended Data Figure 1.** Location of Younger Dryas sub-fossil kauri sites Towai and Finlayson Farm (FIN11). This map was created using CorelDraw X6 (<http://www.coreldraw.com/rw/product/graphic-design-software/>) and modified using Adobe Illustrator CS5 v15.0.0 (http://www.adobe.com/products/illustrator.html).


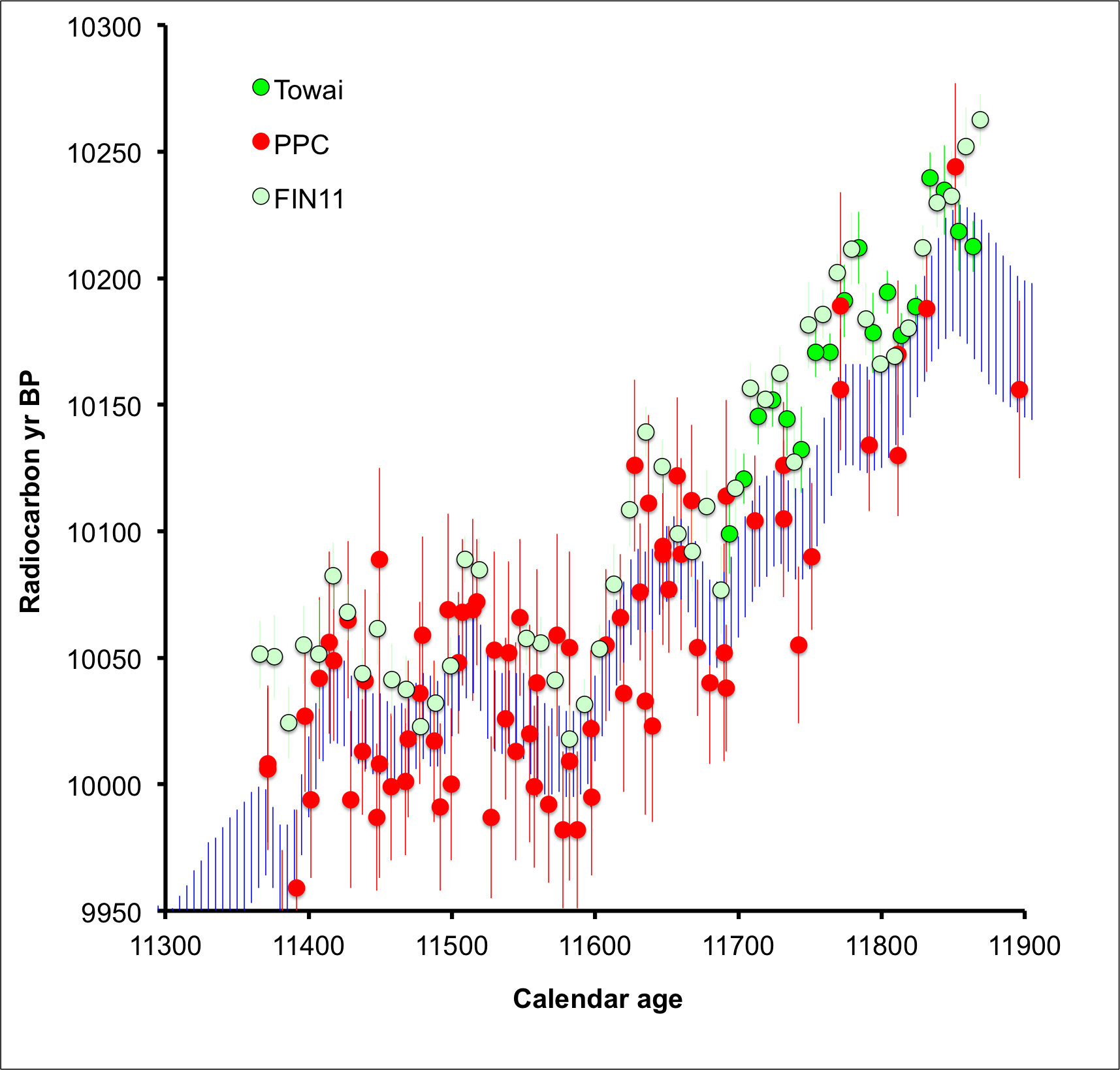


**Extended Data Figure 2.** A match of the youngest part of the Towai chronology (bright green data points) and FIN11 (pale green data points) with PPC data (red) and showing the IntCal13 curve (blue, 1σ error bars). High-precision alignment between ^14^C datasets was undertaken using OxCal 4.2 (refs. ^55,56^). Note, the average number of missing rings for New Zealand kauri is very low (<1%) and false rings are rare^28,54^.


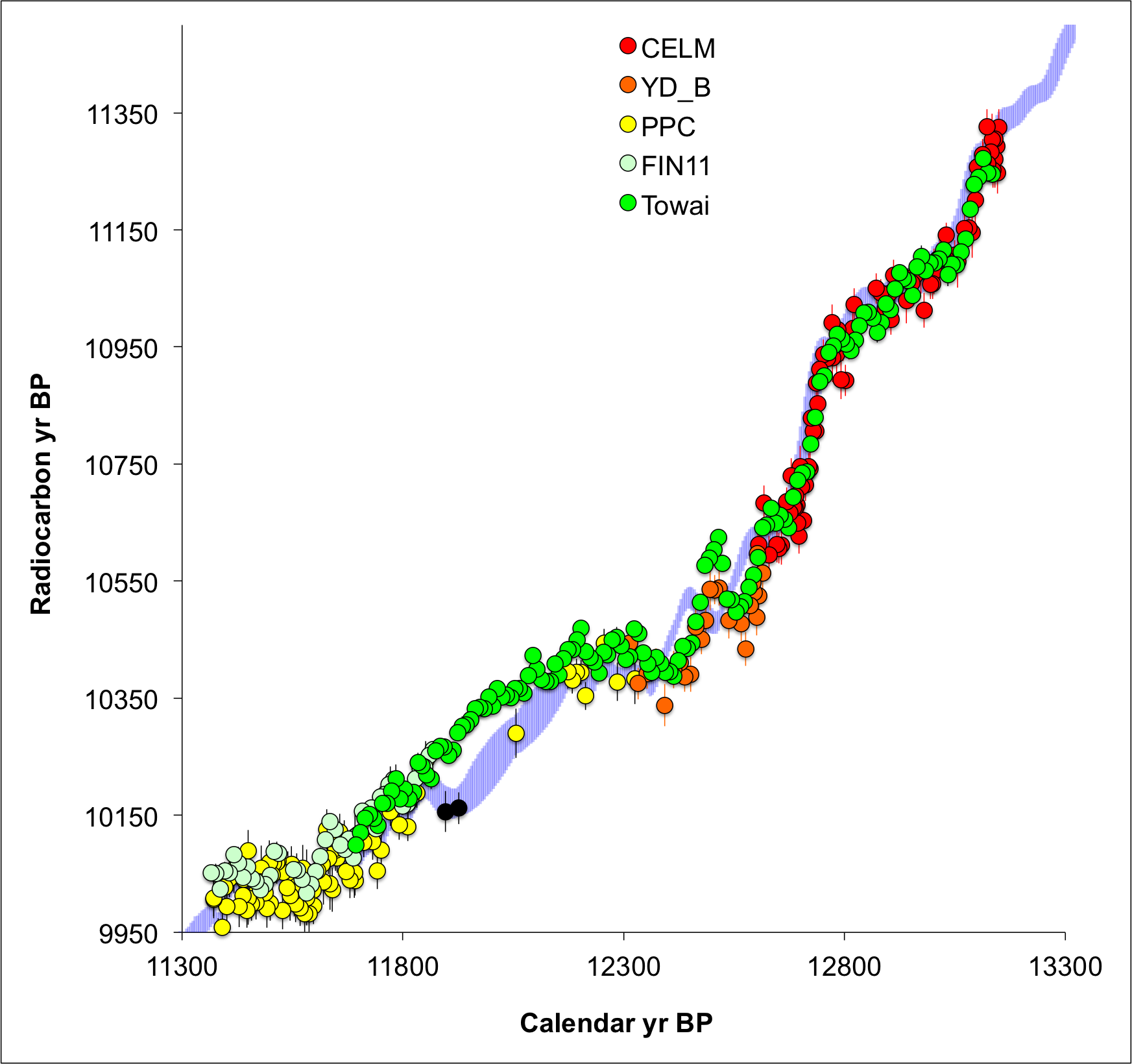


**Extended Data Figure 3.** Northern Hemisphere (PPC, YD_B, CELM)^21,22,24,29^ and Southern Hemisphere (FIN11, Towai) data sets showing the IntCal13 curve (blue, 1σ error bars). Two PPC data points (11,926 and 11,896 cal BP) that appear to be too young are shown in black.


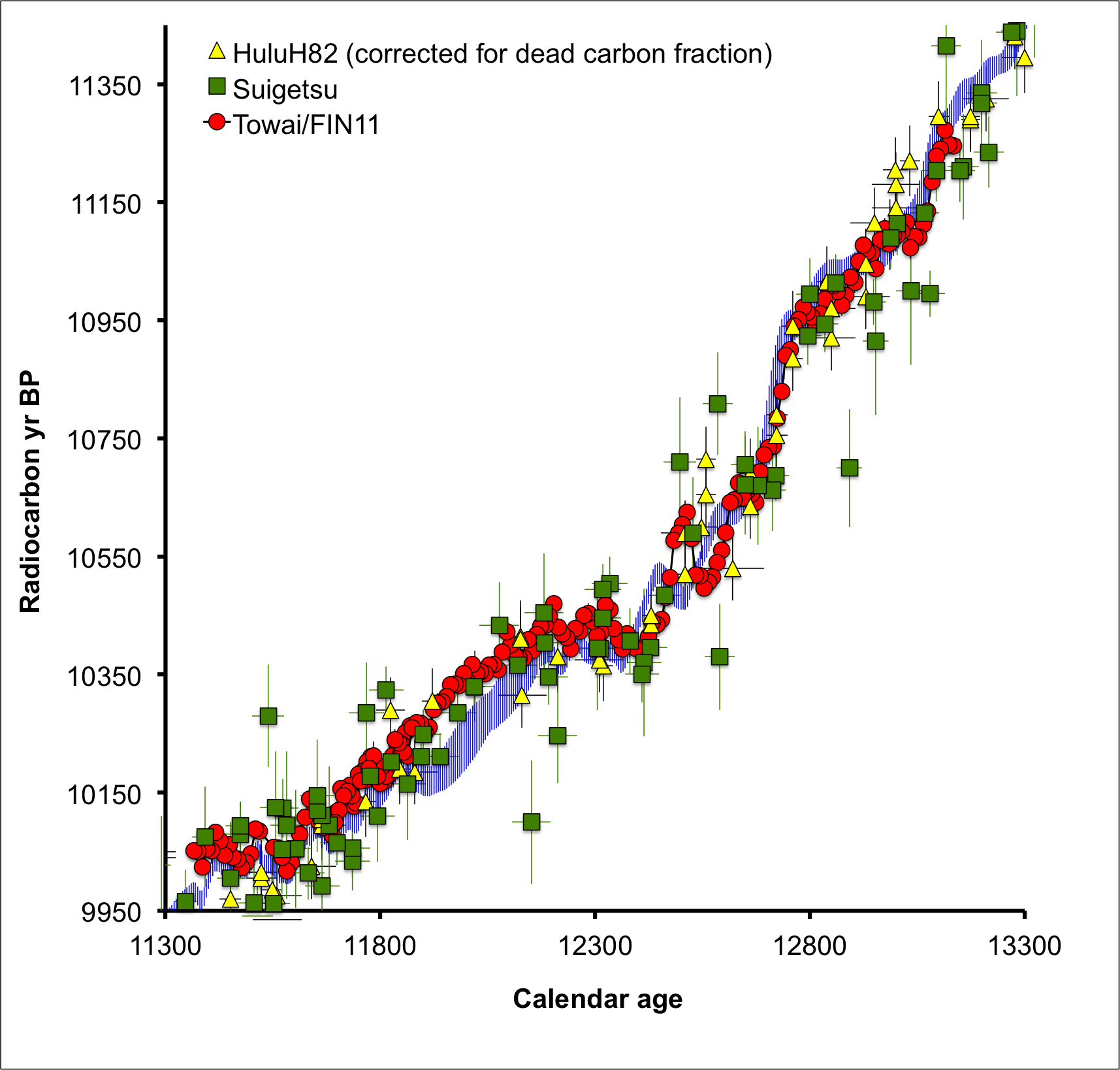


**Extended Data Figure 4.** Hulu H82 (ref. ^30^) and Lake Suigetsu macrofossil^57^ ^14^C dates compared to the Southern Hemisphere (FIN11/Towai) datasets. Also shown is the IntCal13 curve (blue, 1σ errors).


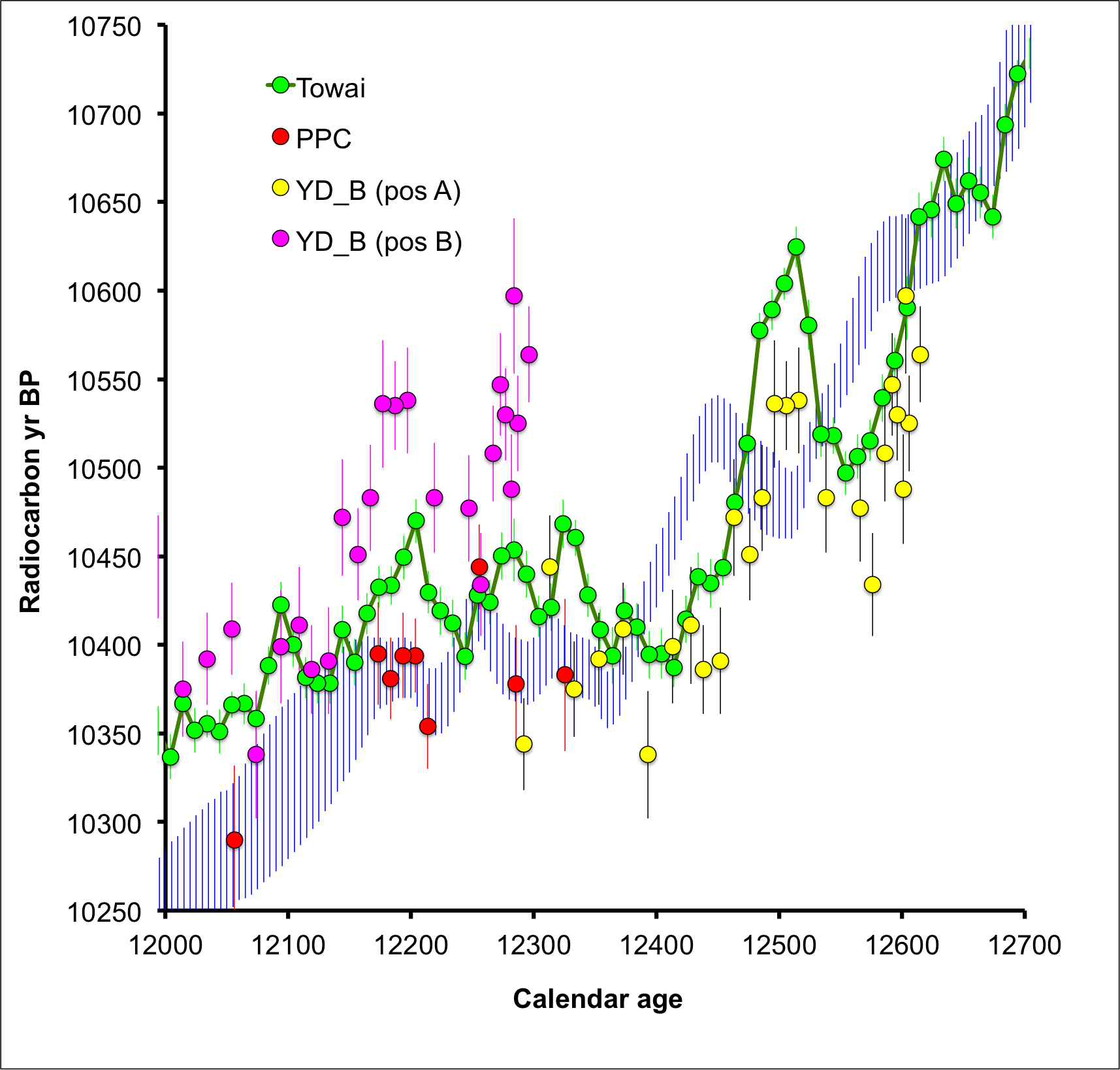


**Extended Data Figure 5.** Two possible locations for the YD_B measurements (pink and yellow) matched against Towai data (green). The 1σ IntCal13 curve is also shown in light blue. Position A (yellow) is considered more likely as it has the higher agreement index (A_c_=202.6%) and the youngest data points conform with the oldest PPC measurements (unlike many of the YD_B data points for position B).


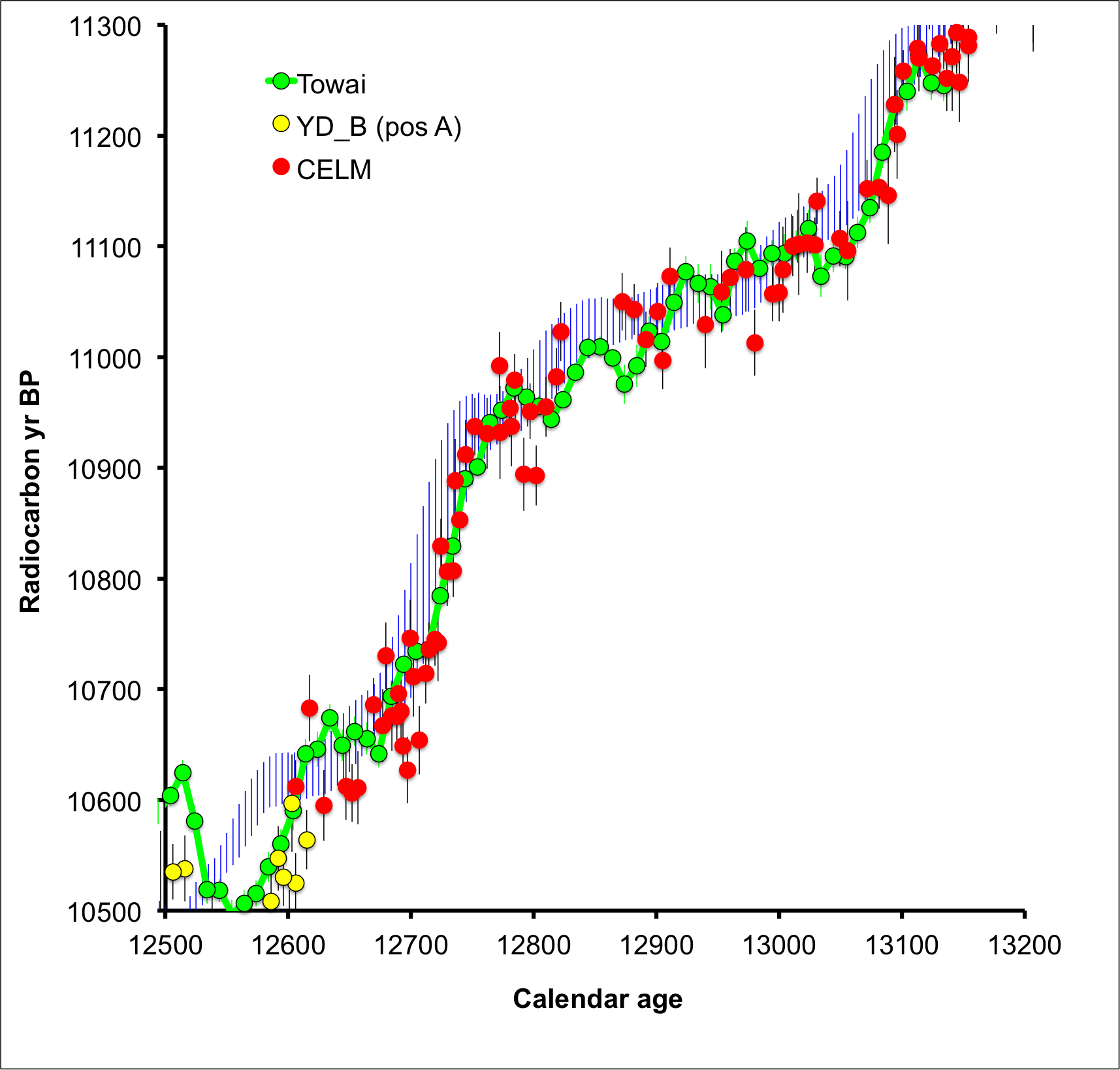


**Extended Data Figure 6.** Match of the youngest 72 CELM measurements (red) with the Towai data (green). The 1σ IntCal13 curve is also shown in light blue. Note, this placement agrees with some studies^22,57^ but disagrees with others^58^.

**Supplementary Tables**

**Supplementary Table 1.** Decadal mean ^14^C values for the Towai chronology – accepted values. Errors calculated according to ref. ^38^.

| Ring  Numbers  (range) | mid-ring  number | Number of analyses accepted per decade | Weighted ^14^C age  (yr BP) | 1σ  error  (E_final_) | Agreement | Number of analyses rejected per decade |
| --- | --- | --- | --- | --- | --- | --- |
| 861-870 | 865.5 | 2 | 11245 | 14 | χ2=0.0 (<3.8) | - |
| 871-880 | 875.5 | 2 | 11248 | 16 | χ2=0.4 (<3.8) | - |
| 881-890 | 885.5 | 2 | 11273 | 14 | χ2=0.8 (<3.8) | - |
| 891-900 | 895.5 | 2 | 11240 | 18 | χ2=0.0 (<3.8) | - |
| 901-910 | 905.5 | 4 | 11228 | 11 | χ2=2.6 (<7.8) | - |
| 911-920 | 915.5 | 2 | 11185 | 14 | χ2=0.1 (<3.8) | - |
| 921-930 | 925.5 | 2 | 11135 | 14 | χ2=0.1 (<3.8) | - |
| 931-940 | 935.5 | 2 | 11113 | 14 | χ2=0.8 (<3.8) | - |
| 941-950 | 945.5 | 4 | 11090 | 11 | χ2=3.2 (<7.8) | - |
| 951-960 | 955.5 | 7 | 11091 | 15 | χ2=11.7 (<12.6) | - |
| 961-970 | 965.5 | 3 | 11073 | 19 | χ2=0.1 (<6.0) | - |
| 971-980 | 975.5 | 3 | 11116 | 18 | χ2=1.9 (<6.0) | - |
| 981-990 | 985.5 | 5 | 11101 | 13 | χ2=4.3 (<9.5) | - |
| 991-1000 | 995.5 | 3 | 11094 | 18 | χ2=1.1 (<6.0) | - |
| 1001-1010 | 1005.5 | 5 | 11094 | 12 | χ2=2.5 (<9.5) | - |
| 1011-1020 | 1015.5 | 5 | 11080 | 14 | χ2=3.8 (<9.5) | - |
| 1021-1030 | 1025.5 | 3 | 11105 | 18 | χ2=0.2 (<6.0) | - |
| 1031-1040 | 1035.5 | 5 | 11087 | 12 | χ2=4.1 (<9.5) | - |
| 1041-1050 | 1045.5 | 5 | 11038 | 15 | χ2=5.0 (<9.5) | - |
| 1051-1060 | 1055.5 | 3 | 11064 | 21 | χ2=2.3 (<6.0) | - |
| 1061-1070 | 1065.5 | 3 | 11067 | 18 | χ2=0.1 (<6.0) | - |
| 1071-1080 | 1075.5 | 5 | 11077 | 14 | χ2=2.9 (<9.5) | - |
| 1081-1090 | 1085.5 | 3 | 11049 | 24 | χ2=3.8 (<6.0) | - |
| 1091-1100 | 1095.5 | 3 | 11014 | 18 | χ2=0.6 (<6.0) | - |
| 1101-1110 | 1105.5 | 5 | 11023 | 13 | χ2=2.6 (<9.5) | - |
| 1111-1120 | 1115.5 | 3 | 10992 | 19 | χ2=1.1 (<6.0) | - |
| 1121-1130 | 1125.5 | 3 | 10975 | 18 | χ2=0.5 (<6.0) | - |
| 1131-1140 | 1135.5 | 10 | 10999 | 8 | χ2=4.3 (<16.9) | - |
| 1141-1150 | 1145.5 | 8 | 11009 | 9 | χ2=5.4 (<14.1) | - |
| 1151-1160 | 1155.5 | 8 | 11008 | 9 | χ2=5.4 (<14.1) | - |
| 1161-1170 | 1165.5 | 10 | 10986 | 9 | χ2=9.2 (<16.9) | - |
| 1171-1180 | 1175.5 | 21 | 10961 | 6 | χ2=14.7 (<31.4) | - |
| 1181-1190 | 1185.5 | 8 | 10944 | 9 | χ2=2.6 (<14.1) | - |
| 1191-1200 | 1195.5 | 9 | 10956 | 9 | χ2=5.0 (<15.5) | - |
| 1201-1210 | 1205.5 | 26 | 10964 | 5 | χ2=13.9 (<37.7) | - |
| 1211-1220 | 1215.5 | 9 | 10972 | 9 | χ2=3.6 (<15.5) | 1 |
| 1221-1230 | 1225.5 | 14 | 10952 | 8 | χ2=17.5 (<22.4) | - |
| 1231-1240 | 1235.5 | 22 | 10941 | 8 | χ2=27.2 (<32.7) | 5 |
| 1241-1250 | 1245.5 | 21 | 10901 | 6 | χ2=21.0 (<31.4) | - |
| 1251-1260 | 1255.5 | 19 | 10890 | 7 | χ2=18.8 (<28.9) | 4 |
| 1261-1270 | 1265.5 | 11 | 10829 | 8 | χ2=5.2 (<18.3) | - |
| 1271-1280 | 1275.5 | 8 | 10784 | 9 | χ2=6.8 (<14.1) | - |
| 1281-1290 | 1285.5 | 10 | 10736 | 9 | χ2=12.3 (<16.9) | - |
| 1291-1300 | 1295.5 | 8 | 10734 | 9 | χ2=4.6 (<14.1) | - |
| 1301-1310 | 1305.5 | 12 | 10722 | 8 | χ2=10.6 (<19.7) | - |
| 1311-1320 | 1315.5 | 8 | 10694 | 12 | χ2=11.2 (<14.1) | - |
| 1321-1330 | 1325.5 | 4 | 10642 | 12 | χ2=2.5 (<7.8) | - |
| 1331-1340 | 1335.5 | 3 | 10655 | 15 | χ2=0.2 (<6.0) | - |
| 1341-1350 | 1345.5 | 5 | 10662 | 13 | χ2=5.6 (<9.5) | - |
| 1351-1360 | 1355.5 | 3 | 10649 | 14 | χ2=0.7 (<6.0) | - |
| 1361-1370 | 1365.5 | 4 | 10674 | 13 | χ2=2.8 (<7.8) | - |
| 1371-1380 | 1375.5 | 3 | 10646 | 16 | χ2=0.9 (<6.0) | - |
| 1381-1390 | 1385.5 | 5 | 10642 | 14 | χ2=7.0 (<9.5) | - |
| 1391-1400 | 1395.5 | 4 | 10590 | 18 | χ2=5.2 (<7.8) | - |
| 1401-1410 | 1405.5 | 4 | 10560 | 13 | χ2=2.8 (<7.8) | - |
| 1411-1420 | 1415.5 | 4 | 10539 | 13 | χ2=0.4 (<7.8) | - |
| 1421-1430 | 1425.5 | 5 | 10515 | 12 | χ2=1.6 (<9.5) | - |
| 1431-1440 | 1435.5 | 5 | 10506 | 13 | χ2=4.4 (<9.5) | - |
| 1441-1450 | 1445.5 | 5 | 10497 | 12 | χ2=1.2 (<9.5) | - |
| 1451-1460 | 1455.5 | 6 | 10518 | 10 | χ2=2.8 (<11.1) | - |
| 1461-1470 | 1465.5 | 4 | 10519 | 13 | χ2=3.2 (<7.8) | - |
| 1471-1480 | 1475.5 | 4 | 10581 | 14 | χ2=2.5 (<7.8) | - |
| 1481-1490 | 1485.5 | 5 | 10625 | 11 | χ2=3.3 (<9.5) | - |
| 1491-1500 | 1495.5 | 8 | 10604 | 9 | χ2=5.2 (<14.1) | - |
| 1501-1510 | 1505.5 | 5 | 10589 | 11 | χ2=0.6 (<9.5) | - |
| 1511-1520 | 1515.5 | 6 | 10578 | 10 | χ2=1.7 (<11.1) | - |
| 1521-1530 | 1525.5 | 5 | 10514 | 12 | χ2=1.2 (<9.5) | - |
| 1531-1540 | 1535.5 | 4 | 10481 | 13 | χ2=0.1 (<7.8) | - |
| 1541-1550 | 1545.5 | 6 | 10444 | 10 | χ2=3.0 (<11.1) | - |
| 1551-1560 | 1555.5 | 4 | 10435 | 14 | χ2=3.1 (<7.8) | - |
| 1561-1570 | 1565.5 | 4 | 10439 | 14 | χ2=1.4 (<7.8) | - |
| 1571-1580 | 1575.5 | 4 | 10414 | 13 | χ2=1.0 (<7.8) | - |
| 1581-1590 | 1585.5 | 6 | 10387 | 11 | χ2=3.3 (<11.1) | - |
| 1591-1600 | 1595.5 | 4 | 10395 | 14 | χ2=2.0 (<7.8) | - |
| 1601-1610 | 1605.5 | 4 | 10394 | 13 | χ2=3.2 (<7.8) | - |
| 1611-1620 | 1615.5 | 4 | 10410 | 13 | χ2=1.1 (<7.8) | - |
| 1621-1630 | 1625.5 | 4 | 10419 | 13 | χ2=2.4 (<7.8) | - |
| 1631-1640 | 1635.5 | 4 | 10394 | 16 | χ2=4.9 (<7.8) | - |
| 1641-1650 | 1645.5 | 5 | 10409 | 11 | χ2=2.9 (<9.5) | - |
| 1651-1660 | 1655.5 | 4 | 10428 | 12 | χ2=1.4 (<7.8) | - |
| 1661-1670 | 1665.5 | 6 | 10461 | 10 | χ2=3.8 (<11.1) | - |
| 1671-1680 | 1675.5 | 4 | 10468 | 14 | χ2=0.7 (<7.8) | - |
| 1681-1690 | 1685.5 | 4 | 10421 | 13 | χ2=2.9 (<7.8) | - |
| 1691-1700 | 1695.5 | 6 | 10416 | 11 | χ2=6.9 (<11.1) | - |
| 1701-1710 | 1705.5 | 4 | 10440 | 13 | χ2=0.6 (<7.8) | - |
| 1711-1720 | 1715.5 | 4 | 10454 | 18 | χ2=5.6 (<7.8) | - |
| 1721-1730 | 1725.5 | 4 | 10450 | 13 | χ2=0.6 (<7.8) | - |
| 1731-1740 | 1735.5 | 4 | 10424 | 12 | χ2=2.0 (<7.8) | - |
| 1741-1750 | 1745.5 | 4 | 10428 | 15 | χ2=4.0 (<7.8) | - |
| 1751-1760 | 1755.5 | 4 | 10394 | 13 | χ2=2.4 (<7.8) | - |
| 1761-1770 | 1765.5 | 4 | 10412 | 13 | χ2=1.7 (<7.8) | - |
| 1771-1780 | 1775.5 | 4 | 10419 | 13 | χ2=0.1 (<7.8) | - |
| 1781-1790 | 1785.5 | 6 | 10430 | 12 | χ2=8.2 (<11.1) | - |
| 1791-1800 | 1795.5 | 4 | 10470 | 12 | χ2=0.8 (<7.8) | - |
| 1801-1810 | 1805.5 | 4 | 10449 | 12 | χ2=2.1 (<7.8) | - |
| 1811-1820 | 1815.5 | 6 | 10433 | 11 | χ2=3.1 (<11.1) | - |
| 1821-1830 | 1825.5 | 4 | 10433 | 12 | χ2=0.3 (<7.8) | - |
| 1831-1840 | 1835.5 | 4 | 10418 | 12 | χ2=2.1 (<7.8) | - |
| 1841-1850 | 1845.5 | 4 | 10390 | 13 | χ2=3.9 (<7.8) | - |
| 1851-1860 | 1855.5 | 4 | 10409 | 14 | χ2=0.8 (<7.8) | - |
| 1861-1870 | 1865.5 | 4 | 10378 | 12 | χ2=1.0 (<7.8) | - |
| 1871-1880 | 1875.5 | 4 | 10378 | 12 | χ2=0.4 (<7.8) | - |
| 1881-1890 | 1885.5 | 4 | 10382 | 12 | χ2=0.4 (<7.8) | - |
| 1891-1900 | 1895.5 | 4 | 10400 | 12 | χ2=1.9 (<7.8) | - |
| 1901-1910 | 1905.5 | 4 | 10423 | 13 | χ2=0.2 (<7.8) | - |
| 1911-1920 | 1915.5 | 6 | 10388 | 11 | χ2=4.3 (<11.1) | - |
| 1921-1930 | 1925.5 | 4 | 10358 | 14 | χ2=0.4 (<7.8) | - |
| 1931-1940 | 1935.5 | 5 | 10367 | 12 | χ2=1.6 (<9.5) | - |
| 1941-1950 | 1945.5 | 13 | 10366 | 7 | χ2=12.5 (<21.0) | - |
| 1951-1960 | 1955.5 | 4 | 10351 | 13 | χ2=0.4 (<7.8) | - |
| 1961-1970 | 1965.5 | 13 | 10355 | 8 | χ2=13.9 (<21.0) | - |
| 1971-1980 | 1975.5 | 4 | 10352 | 13 | χ2=2.6 (<7.8) | - |
| 1981-1990 | 1985.5 | 6 | 10367 | 10 | χ2=3.3 (<11.1) | - |
| 1991-2000 | 1995.5 | 4 | 10337 | 13 | χ2=1.5 (<7.8) | - |
| 2001-2010 | 2005.5 | 4 | 10352 | 14 | χ2=1.7 (<7.8) | - |
| 2011-2020 | 2015.5 | 5 | 10332 | 14 | χ2=1.5 (<9.5) | - |
| 2021-2030 | 2025.5 | 6 | 10334 | 11 | χ2=6.5 (<11.1) | - |
| 2031-2040 | 2035.5 | 4 | 10332 | 13 | χ2=1.7 (<7.8) | - |
| 2041-2050 | 2045.5 | 4 | 10313 | 13 | χ2=1.0 (<7.8) | - |
| 2051-2060 | 2055.5 | 4 | 10304 | 13 | χ2=2.1 (<7.8) | - |
| 2061-2070 | 2065.5 | 4 | 10302 | 16 | χ2=4.4 (<7.8) | - |
| 2071-2080 | 2075.5 | 4 | 10291 | 14 | χ2=1.9 (<7.8) | - |
| 2081-2090 | 2085.5 | 4 | 10261 | 13 | χ2=1.3 (<7.8) | - |
| 2091-2100 | 2095.5 | 4 | 10252 | 14 | χ2=2.6 (<7.8) | - |
| 2101-2110 | 2105.5 | 4 | 10267 | 13 | χ2=2.1 (<7.8) | - |
| 2111-2120 | 2115.5 | 6 | 10268 | 11 | χ2=1.7 (<11.1) | - |
| 2121-2130 | 2125.5 | 3 | 10260 | 12 | χ2=1.6 (<6.0) | - |
| 2131-2140 | 2135.5 | 3 | 10212 | 18 | χ2=3.7 (<6.0) | - |
| 2141-2150 | 2145.5 | 3 | 10219 | 14 | χ2=2.8 (<6.0) | - |
| 2151-2160 | 2155.5 | 3 | 10235 | 14 | χ2=1.7 (<6.0) | - |
| 2161-2170 | 2165.5 | 4 | 10240 | 17 | χ2=6.6 (<7.8) | - |
| 2171-2180 | 2175.5 | 5 | 10189 | 11 | χ2=5.3 (<9.5) | - |
| 2181-2190 | 2185.5 | 4 | 10178 | 14 | χ2=4.6 (<7.8) | - |
| 2191-2200 | 2195.5 | 4 | 10194 | 11 | χ2=2.2 (<7.8) | - |
| 2201-2210 | 2205.5 | 3 | 10178 | 14 | χ2=0.7 (<6.0) | - |
| 2211-2220 | 2216.5 | 1 | 10212 | 25 |  | - |
| 2221-2230 | 2225.5 | 6 | 10191 | 12 | χ2=8.6 (<11.1) | - |
| 2231-2240 | 2235.5 | 4 | 10171 | 12 | χ2=2.9 (<7.8) | - |
| 2241-2250 | 2245.5 | 3 | 10171 | 15 | χ2=1.3 (<6.0) | 1 |
| 2251-2260 | 2255.5 | 4 | 10132 | 12 | χ2=0.9 (<7.8) | - |
| 2261-2270 | 2265.5 | 5 | 10144 | 12 | χ2=5.3 (<9.5) | - |
| 2271-2280 | 2275.5 | 3 | 10152 | 12 | χ2=1.8 (<6.0) | - |
| 2281-2290 | 2285.5 | 4 | 10145 | 12 | χ2=0.9 (<7.8) | - |
| 2291-2300 | 2295.5 | 3 | 10121 | 12 | χ2=1.1 (<6.0) | - |
| 2301-2310 | 2305.5 | 3 | 10099 | 15 | χ2=3.1 (<6.0) | - |

**Supplementary Table 2.** Decadal mean ^14^C values for the tree FIN11 – accepted values.

| Ring  Numbers  (range) | mid-ring  number | Number of analyses per decade | Weighted ^14^C age  (yr BP) | 1σ  error  (E_final_) | Agreement | Number of analyses rejected per decade |
| --- | --- | --- | --- | --- | --- | --- |
| 21-30 | 25.5 | 5 | 10263 | 10 | χ2=2.8 (<9.5) | - |
| 31-40 | 35.5 | 2 | 10252 | 16 | χ2=0.0 (<3.8) | - |
| 41-50 | 45.5 | 2 | 10233 | 18 | χ2=0.0 (<3.8) | - |
| 51-60 | 55.5 | 11 | 10230 | 10 | χ2=13.7 (<18.3) | - |
| 61-70 | 65.5 | 8 | 10212 | 9 | χ2=5.4 (<14.1) | - |
| 71-80 | 75.5 | 11 | 10180 | 9 | χ2=10.7 (<18.3) | - |
| 81-90 | 85.5 | 10 | 10169 | 9 | χ2=7.0 (<16.9) | - |
| 91-100 | 95.5 | 3 | 10166 | 16 | χ2=0.3 (<6.0) | - |
| 101-110 | 105.5 | 3 | 10184 | 14 | χ2=1.9 (<6.0) | - |
| 111-120 | 115.5 | 3 | 10212 | 14 | χ2=1.0 (<6.0) | - |
| 121-130 | 125.5 | 14 | 10202 | 7 | χ2=11.1 (<22.4) | - |
| 131-140 | 135.5 | 11 | 10186 | 10 | χ2=12.5 (<18.3) | - |
| 141-150 | 145.5 | 3 | 10181 | 17 | χ2=2.9 (<6.0) | - |
| 151-160 | 155.5 | 3 | 10127 | 14 | χ2=1.3 (<6.0) | - |
| 161-170 | 165.5 | 6 | 10162 | 11 | χ2=6.0 (<11.1) | - |
| 171-180 | 175.5 | 9 | 10152 | 11 | χ2=12.4 (<15.5) | - |
| 181-191 | 186.0 | 13 | 10157 | 10 | χ2=11.6 (<21.0) | 2 |
| 192-201 | 196.5 | 3 | 10117 | 16 | χ2=0.9 (<6.0) | - |
| 202-211 | 206.5 | 3 | 10077 | 17 | χ2=2.9 (<6.0) | - |
| 212-221 | 216.5 | 3 | 10110 | 14 | χ2=0.2 (<6.0) | - |
| 222-231 | 226.5 | 3 | 10092 | 16 | χ2=1.8 (<6.0) | - |
| 232-241 | 236.5 | 3 | 10099 | 19 | χ2=3.7 (<6.0) | - |
| 242-253 | 247.5 | 6 | 10126 | 11 | χ2=5.3 (<11.1) | - |
| 254-264 | 259.0 | 5 | 10139 | 10 | χ2=3.6 (<9.5) | - |
| 265-276 | 270.5 | 3 | 10108 | 14 | χ2=0.1 (<6.0) | - |
| 277-286 | 281.5 | 3 | 10079 | 16 | χ2=0.1 (<6.0) | - |
| 287-296 | 291.5 | 8 | 10053 | 10 | χ2=8.2 (<14.1) | - |
| 297-307 | 302.0 | 5 | 10032 | 10 | χ2=3.7 (<9.5) | - |
| 308-317 | 312.5 | 5 | 10018 | 13 | χ2=7.2 (<9.5) | - |
| 318-327 | 322.5 | 5 | 10041 | 11 | χ2=3.2 (<9.5) | - |
| 328-337 | 332.5 | 5 | 10056 | 10 | χ2=1.6 (<9.5) | - |
| 338-347 | 342.5 | 5 | 10058 | 11 | χ2=2.1 (<9.5) | - |
| 371-380 | 375.5 | 5 | 10085 | 13 | χ2=5.1 (<9.5) | - |
| 381-390 | 385.5 | 8 | 10089 | 9 | χ2=8.6 (<14.1) | - |
| 391-400 | 395.5 | 4 | 10047 | 11 | χ2=3.2 (<7.8) | - |
| 401-411 | 406.0 | 3 | 10032 | 16 | χ2=0.2 (<6.0) | - |
| 412-421 | 416.5 | 3 | 10023 | 14 | χ2=0.2 (<6.0) | - |
| 422-431 | 426.5 | 3 | 10037 | 13 | χ2=1.3 (<6.0) | - |
| 432-441 | 436.5 | 3 | 10041 | 14 | χ2=0.4 (<6.0) | - |
| 442-451 | 446.5 | 3 | 10062 | 14 | χ2=0.1 (<6.0) | - |
| 452-462 | 457.0 | 6 | 10044 | 10 | χ2=5.0 (<11.1) | - |
| 463-472 | 467.5 | 6 | 10068 | 11 | χ2=5.4 (<11.1) | - |
| 473-482 | 477.5 | 3 | 10082 | 13 | χ2=0.4 (<6.0) | - |
| 483-492 | 487.5 | 3 | 10051 | 22 | χ2=3.7 (<6.0) | - |
| 493-503 | 498.0 | 3 | 10055 | 15 | χ2=2.8 (<6.0) | - |
| 504-513 | 508.5 | 3 | 10024 | 14 | χ2=0.8 (<6.0) | - |
| 514-523 | 518.5 | 3 | 10050 | 16 | χ2=3.1 (<6.0) | - |
| 524-533 | 528.5 | 3 | 10051 | 13 | χ2=1.4 (<6.0) | - |
